# Pancreatic involvement in small cell lung cancer

**DOI:** 10.2478/raon-2013-0022

**Published:** 2014-01-22

**Authors:** Ugur Gonlugur, Arzu Mirici, Muammer Karaayvaz

**Affiliations:** 1 Department of Chest Diseases, School of Medicine, Canakkale Onsekiz Mart University, 17100 Canakkale, Turkey; 2 Department of General Surgery, School of Medicine, Canakkale Onsekiz Mart University, 17100 Canakkale, Turkey

**Keywords:** lung neoplasms, carcinoma, small cell, neoplasm metastasis, pancreas

## Abstract

**Background:**

Few data are available concerning incidence, clinical picture, and prognosis for pancreatic metastases of small cell lung carcinoma. In this paper we review the related literature available in English language.

**Conclusions:**

Although pancreatic metastases are generally asymptomatic, they can rarely produce clinical symptoms or functional abnormalities. The widespread use of multi-detector computerised tomography (CT) in contemporary medical practice has led to an increased detection of pancreatic metastases in oncology patients. Tissue diagnosis is imperative because radiological techniques alone are incapable of differentiating them from primary pancreatic tumours. Pancreatic metastases occur in the relative end stage of small cell lung cancer. The main complications of these lesions, although rare, are acute pancreatitis and obstructive jaundice. Early chemotherapy can provide a survival benefit even in patients with mild acute pancreatitis or extrahepatic biliary obstruction.

## Introduction

With the improvement of imaging techniques, pancreas metastases are much more frequent than commonly appreciated. There were limited data about pancreas metastases of small cell lung cancer. Few guidelines are available on the appropriate management of such cases. The aim of this study is to review the pancreatic involvement due to small cell carcinoma of the lung.

## Methods

A computerised research of literature (English language only) was performed to identify the relevant articles dealing with the subject. Using the medical subject headings and terms “pancreas metastasis or pancreatic metastases”, “small cell”, and “lung” we reviewed *PubMed* and *Web of science* databases between 1970 and 2012. The selection criteria included patients with published demographic and clinical data regarding the treatment for cancer metastasized to pancreas. Exclusion criteria for this research included publications of patients’ data in non-English language, patients without previous translation and lack of relevance of the analysis of pancreatic metastasis. Each paper was inspected and the reference lists of the selected articles were also screened systematically for additional studies of interest. More than 20 cases of primary small cell carcinoma of the pancreas were excluded.^1^–^6^ Additionally, some cases with small cell cancer in the studies were analysed just to some extent because they did not describe the detailed clinical findings 7–18 Information regarding patient presentation, site of primary neoplasia, characteristics of metastasis in the pancreas, treatment, and patient demographics were summarised using descriptive statistics. All tests were performed using SPSS 15.0 for Windows.

## Frequency of pancreatic metastasis

The precise prevalence of pancreatic metastasis is not clear. The reported frequency of pancreatic metastases was 1.6% in 4955 adult autopsy cases.^9^ This rate was 5.9% in 1740 autopsies of another study.^11^ Pancreas metastasis has been observed in 25 (2.1%) patients of 1172 pancreatic endoscopic ultrasound-guided fine-needle aspiration (EUS-FNA).^19^ This rate was, respectively 10.7%, 2.4%, and 0.7% in different studies.^18^,^20^,^21^ Because the radiological examination is generally conducted in an early stage, the occurrence of pancreatic metastases in cancer patients undergoing computerised tomography (CT) should be significantly lower than in patients who have died of cancer.^22^ On the other hand, the metastases have been only detected at the microscopic level in 25% of the patients in autopsy series.^9^

The primary site of metastatic pancreatic tumour differs according to the study. The most common site can be lung carcinoma, renal cell carcinoma, colon carcinoma, or stomach carcinoma.^9^–^13^,^15^,^16^,^18^,^19^,^21^–^27^ Mesa *et al.* reviewed all secondary tumours of pancreas in literature by Medline between 1966 and 2003.^18^ Lungs (18.7%), gastrointestinal tract (17.7%), kidneys (16.3%), breasts (10.6%), and lymphomas (7.9%) were the most frequent secondary neoplasms in 699 cases. The reported incidence by site of origin reflects the overall incidence of cancers in the general population. However, there are a disproportionately high number of reports of metastases from renal cell carcinoma. Another review in 2004 showed that the predominant primary tumour was renal cell carcinoma in 150 cases (45%), followed by lung carcinoma in 49 cases (14.7%), breast carcinoma in 25 cases (7.5%), and colon cancer in 22 cases (6.6%).^28^ Sweeney *et al.* searched PubMed using the terms “pancreatic metastasis” and “cancer metastatic to the pancreas” after 2004.^29^ They found that the most common primary tumour site was kidney (70.5%), followed by breast (6.8%), lung (5.9%), and colorectal (5.5%) for 220 patients. It has been suggested that pancreatic metastases may arise from local lymphatic or venous dissemination because the pancreas and kidneys are located close to one another within the retroperitoneal compartment.^25^

Smith reviewed the incidence of metastasis to the hepatic-pancreatic-biliary axis in primary carcinoma of the lung.^30^ A total of 679 (10%) patients were found in 6807 autopsies between 1929 and 1975. On searching medical records of 850 patients with lung cancer, 26 (3.1%) cases were identified with pancreatic metastasis.^31^ Fluorine-18 fluorodeoxyglucose positron emission tomography (FDG-PET) is a powerful tool for oncology imaging. Pancreatic metastases were found in 1.9% of 573 cases with lung cancer by FDG-PET/CT study.^7^ However, only one of the pancreatic lesions had histological confirmation in this study. Because primary pancreatic cancer and pancreatitis also show FDG accumulation, the actual rate of metastasis to pancreas in lung cancer may be lower than 1.9 percent. A pathological confirmation is sometimes considered unjustifiable both from the patient’s and an economic standpoint. This problem also alters the true incidence of metastatic disease of the pancreas.

The most common histological type of lung cancer that is related to pancreatic metastasis was reported as small cell lung cancer, followed by large cell carcinoma, squamous cell carcinoma, and adenocarcinoma.^12^,^30^,^32^–^35^ For metastasis-induced acute pancreatitis patients with lung cancer, the rate of small cell carcinoma to non-small cell carcinoma was 15/18 in one study and 5/15 in another study.^8^,^36^

## Clinical picture

The symptoms produced by metastasis to the pancreas are variable. Most patients (50–83%) are free of organ-specific complaints (abdominal pain, jaundice, abdominal discomfort, weight loss) when the metastasis are detected incidentally on CT during periodic clinical surveillance or follow-up. 25,37,38 Metastases can directly invade pancreatic ductal epithelium and thus may clinically mimic primary pancreatic adenocarcinoma.^37^ Organ-related clinical symptoms were observed in only 6 of our 18 patients.^10^ In a review of 220 patients, the most common presenting symptoms were jaundice (25.2%), and abdominal pain (11.4%). In their survey, 27.6% of the patients were asymptomatic.^29^ Male/female ratio was 48/22 in our survey. Mean age was 59±10 (range 37–83). The most common location was the left lung (59%).

### Time of metastasis

Pancreatic metastasis generally constitutes a late manifestation of widespread disseminated metastasis in cancer patients.^39^ In Mayo Clinic series, only 27 of 1357 patients (2%) with solitary pancreatic masses had secondary pancreatic tumour.^26^ For small cell lung cancer, all ten patients in autopsy series and all eight patients in clinical series are presented as disseminated disease.^9^,^38^ In addition to these 18 patients, another 49 patients (Table 1, 2, and 3) also had other metastases, but 10 had solitary pancreatic metastasis. Consequently, the rate of solitary pancreatic metastasis to disseminated metastases was 10/67. The overall survival of these patients may be lower because of the extensive disease. The presence of pancreatic metastases indicates the visceral widespread and is of ominous prognostic importance. Median survival for 4 patients who had not other metastasis was 8.25 months but 1.7 months for 29 patients with extensive disease in our survey.

### Jaundice due to small cell lung cancer

Lung cancer can cause biliary tract obstruction by metastasising to lymph nodes in the porta hepatis, pancreas or hepatic parenchyma.^40^ Johnson *et al*. reported that 12 (9.6%) among 125 patients presented with hyperbilirubinaemia with small cell lung cancer at diagnosis.^14^ Five of these patients had pancreatic metastasis resulting in extrahepatic biliary obstruction, and seven had diffuse hepatic metastasis without extrahepatic biliary obstruction. When compared to extrahepatic obstruction, the resolution of jaundice is more difficult in patients with diffuse hepatic metastasis. Of 19 patients reported by Smith, eight (66%) patients with small cell lung cancer had evidence of extrahepatic bile duct obstruction.^30^ Tumours located in the head of the gland usually cause obstructive jaundice (Table 1), whereas tumours in the body and tail of the pancreas may lead to dilatation of Wirsung’s duct.^62^ Palliative treatment by surgical biliary bypass and postoperative chemotherapy are indicated in such patients even though the patients had diffuse hepatic metastases.^40^ This approach may produce extended survival exceeding 4 months. Table 1 shows that patients treated only with surgery survive several weeks. Median survival for 6 patients treated with chemotherapy was 10.5 months but 2 weeks for 5patients treated with conservative treatment in our survey.

### Acute pancreatitis due to small cell lung cancer

Associated pathological findings in cases with secondary pancreatic tumours were reported as fat necrosis (19%), chronic pancreatitis (11%), acute pancreatitis (8%), and acute terminal pancreatitis (6%).^11^ Although gastric cancer has been noted the most common nonpancreatic tumour associated with pancreatitis, small cell lung carcinoma has been reported to be the most common (6 of 10 patients) lung tumour causing metastasis-induced acute pancreatitis.^46^,^47^ Some lung cancer patients with metastasis-induced acute pancreatitis, especially patients with small cell lung cancer, have no obvious symptoms, and were easily underdiag-nosed.^36^

In 1973, Levine and Danovitch first described a patient with small cell lung cancer in whom acute pancreatitis developed during the progression of the malignancy.^48^ In a series of 40 consecutive cases of small cell cancer of the lung during the period from August 1974 to August 1977, three cases of metastasis-induced acute pancreatitis were discovered. This represented an occurrence rate of 7.5 per cent.^49^ Although the frequency of the clinical picture was 3.3% in another series, the rate of metastasis-induced acute pancreatitis was reported as 0.12% in the largest study consisting of 802 patients.^47^,^50^

There are several mechanisms by which a meta-static tumour might induce acute pancreatitis. The first is the obstruction of the pancreatic duct by metastases or peripancreatic compression secondary to regional lymph nodes. This can lead to the activation of the pancreatic proteases, resulting in subsequent autolysis. The second is the vascular compromise by neoplastic destruction.^10^,^49^ In a series, almost 40% of patients with metastatic lesions had obstruction of the main pancreatic duct.^22^ More than half of these patients had neoplasms in the pancreatic head with associated biliary ductal obstruction.

In some patients, the possible causes of acute pancreatitis such as alcohol consumption, gallstone disease, or the use of antineoplastic agents such as ifosfamide, vinorelbine, and cisplatin can be found.^8^,^33^,^46^–^53^ On the other hand, pathological investigation showed no evidence of metastatic tumour in some patients.^52^ This finding suggested that acute pancreatitis could be a paraneoplastic syndrome. Pancreatic metastasis could be confirmed by pancreatic biopsy, but it is often difficult to obtain a tissue diagnosis in these seriously ill patients due to high morbidity and false negative rate of biopsy.^46^ In our survey, thirteen patients (7 of them had post-mortem diagnosis) had tissue confirmation of pancreatic metastasis, but 13 patients had not had a pathological sampling of pancreatic tumour (Table 2). Interestingly, we found left lung predominance (11/7) in patients with acute pancreatitis. It is necessary to investigate the lymphatic communication between the left lung and pancreas.

Treatment for tumour-induced acute pancreatitis is initially supportive. In patients with mild pancreatitis, chemotherapy may favourably influence the recovery from pancreatitis. However, chemotherapy is poorly tolerated by patients with severe pancreatitis and it is therefore inadvisable in patients with high (>3) Ranson scores.^50^ Of the two cases reported, one patient receiving supportive treatment died in one month, and the other patient survived only seven weeks in spite of palliative abdominal radiotherapy.^48^ There was only one study that investigated the prognostic factors for metastasis-induced acute pancreatitis patients with lung cancer.^36^ Chemotherapy and performance status have been found to be significant factors for survival. In our survey median survival for 6 patients treated with chemotherapy was 6 months, but two weeks for 5 patients treated with conservative treatment.

## Radiographic features

Lesions are primarily diagnosed on radiographic techniques. Imaging techniques are able to delineate the mass and evaluate its size and lymph node status, as well as the extent of vascular invasion. Pancreatic metastasis must be distinguished from local invasion of malignant gastrointestinal tumours. Although abdominal ultrasound is the first-line imaging, CT is the most frequently used method for studying pancreatic cancer.^67^ Ultrasonography also helps to distinguish the aetiology of the common causes of pancreatitis and it is important to exclude cholelithiasis if chemotherapy is to be considered.^54^

The sensitivity of abdominal ultrasound in the detection of all tumours in the pancreas was found as 89% in a prospective study.^68^ The sonographic appearance of metastasis appeared as homogenous, well-demarcated solid lesion with a more hypoechoic internal structure than the pancreatic parenchyma. The pancreatic duct is usually not dilated. Although metastases can be hypovascular or hypervascular depending on the primary malignancy, a pancreatic mass rich in blood flow, as indicated by colour Doppler ultrasound, is more likely to be a metastasis than a primary pancreatic carcinoma.^39^,^69^,^70^ The metastatic lesion from a renal cell carcinoma is usually highly vascular, and contrast-enhanced ultrasound or angiography is very useful for diagnosis.^12^ Endoscopic ultrasound can show invisible metastases in abdominal ultrasound.^55^ Interestingly, abdominal ultrasound can show invisible metastases in CT.^56^ Endoscopic ultrasound features of primary pancreatic cancer and metastatic lesion were similar with respect to tumour size, echogenicity, or consistency. Although endoscopic ultrasound is probably superior for detection of small tumours, helical CT appears equal to or perhaps even better than endoscopic ultrasound for staging pancreatic neoplasms.^20^

For 12 patients with pancreas metastasis, abdominal ultrasonography had reported hypoechoic lesion in 6 patients, a hypertrophic aspect involving the head of the pancreas with dilatation of the main pancreatic duct in one patient, and an aspect suggestive of acute pancreatitis in one patient.^15^ In their study, abdominal CT was performed in 19 patients and revealed tumours in 13 patients, increased pancreatic volume in 5 patients, and an aspect compatible with pancreatitis in one patient. CT technology has improved greatly over the last decade. For primary pancreatic cancer, an accuracy of 99% has been reported for prediction of vascular invasion by tumour with multi-detector helical CT (MDCT).^71^ Although the sensitivity of modern MDCT scanners in demonstrating pancreatic metastasis is still not known, MDCT with 2D multi-planar reconstructions (MPR) and three-dimensional rendering allows detection of lesions that are not visible on routine CT images.^53^ In a study of 11 cases, nearly 30% of pancreatic metastases were missed on routine transaxial images and detected only on thin slice or MPR images.^7^ There was only one study that investigated the diagnostic performance of MDCT in pancreatic metastases.^67^ All metastatic lesions in 17 patients, ranged between 8 and 40 mm, were demonstrated by MDCT.

Precontrast CT images of the pancreas show either isodense or hypodense lesions when compared to the normal parenchyma, with rare regions of calcification.^69^ Solitary metastases typically demonstrate areas of low attenuation (probably indicative for necrotic foci) on contrast enhanced CT, while the diffuse form of metastasis remains isodense following intravenous contrast medium.^10^,^41^ A peripheral rim of enhancement after intravenous contrast medium is usually demonstrated. Rim enhancement is especially common in lesions larger than 1.5 cm in size, whereas smaller tumours display a more uniform vascular pattern.^41^ Although some authors did not observe a correlation between tumour size and pattern of enhancement, they also found a peripheral enhancement in 73% of the patients.^24^ The peripheral enhancement of tumour tissue observed in many cases reflects a degree of vascular perfusion that is not typical of primary pancreatic adenocarcinoma, whereas ductal adenocarcinoma of the pancreas typically appears as a uniformly nonenhancing mass at contrast-enhanced.^22^,^24^

CT can occasionally fail to show subtle differences in attenuation between normal pancreatic tissue and non-necrotic or non-cystic neoplastic tissue.^23^ Of 34 patients with autopsy-proven secondary tumour, pancreatic abnormalities were documented on non-helical abdominal CT only in 18 (53.8%) cases. Initial CT of the pancreas can be normal in appearance in patients with metastasis.^10^ Lymphatic metastasis of the pancreas can occur without any tumour formation, what is often seen in stomach carcinoma.^11^

On magnetic resonance imaging, pancreatic metastases typically appear hypointense and well circumscribed compared with normal gland tissue on unenhanced T1-weighted images, both with and without fat saturation. Following intravenous gadopentetate dimeglumine administration, a rim of enhancement is usually visible in larger lesions and homogenous enhancement is typically demonstrated in smaller tumours. On T2-weighted images, the lesions are slightly heterogeneous and moderately hyperintense. Hypointense nodules are sometimes visible on T2-weighted images, especially in the diffusely enlarged type.^38^,^41^

A definitive diagnosis requires a pathologic examination of a biopsy or surgical specimen. Endoscopic ultrasound-guided fine-needle aspiration has become the preferred method for cytologic-tissue acquisition of pancreatic masses, especially following equivocal CT imaging.^69^ Nevertheless, the histological distinction between primary pancreatic cancer and metastatic tumour is sometimes difficult to establish.^15^

## Radiological pattern of metastasis

The majority of overall metastatic lesions were found in the head of the pancreas.^16^,^20^,^29^,^32^ For lung cancer, some authors reported that the majority of metastatic lesions were observed in the body or the head of the pancreas.^33^,^38^ However, our review showed that the most common macroscopic location is the head (76%) of the gland for small cell lung cancer (Table 1, Table 2, and Table 3). The body was the second most common site (15%) for solitary involvements.

Three patterns of metastatic involvement of the pancreas have been described. The first and most common, reported in 50–80% of cases, is that of a single localised mass.^19^ A second pattern of diffuse pancreatic enlargement has been reported in 15–44% of cases. Diffuse enlargement is characterised by a diffuse infiltration of malignant cells along the interlobular septa, which causes the destruction of large parts of the pancreatic lobules.^23^ The gland has homogenous density in CT appearance. The third pattern is multiple pancreatic nodules (10–44%), represented by many small nodules which can coalesce occasionally into larger masses.^12^,^22^,^23^,^37^ The attenuation of the neoplastic nodules may be variable.^49^

Solitary nodule was the usual pattern of pancreatic metastasis, both for overall tumours and for lung cancer.^16^,^22^–^25^,^31^ Although the most common cause of diffuse infiltrative type of metastasis reported is small cell carcinoma of the lung [10], in our survey of 50 patients with small cell lung cancer, we observed a solitary nodule in 29 patients (57%), multiple nodules in 14 patients (27%), and diffuse enlargement in 8 (16%) the patients. Radiological pattern can be related to the invasiveness of metastatic carcinoma.^10^ Interestingly, we found that diffuse enlargement is the most common pattern (50%) in patients with acute pancreatitis (Table 2). On the other hand, the most common type of metastasis was diffuse involvement in histological examination.^10^

## Conclusions

The frequency of pancreatic metastasis varies between 1.6% and 10.6% in autopsy studies. Secondary tumours constitute 3–17% of all pancreatic tumours in clinical studies, but 43–65% in autopsy studies. The most common pattern for small cell lung cancer is a single localised mass in the head of the gland. The primary tumour is generally found in the left lung. Less than 15% of the patients had solitary pancreatic metastasis but other extensive disease. In spite of the advent of other imaging modalities, CT is still the gold standard for the evaluation of pancreatic pathology. In the patients with jaundice, systemic chemotherapy might resolve the obstruction with symptomatic relief for the patient. Metastasis-induced acute pancreatitis can be a lethal event, and the diagnosis should be considered in any patient with known small cell carcinoma in whom an acute abdomen develops. Because of the aggressive nature of the tumour, all patients who had an appropriate performance status should receive systemic chemotherapy. The prognosis is better in patients with solitary pancreatic metastasis.

## Figures and Tables

**TABLE 1.** The characteristics of the patients with pancreatic metastasis-induced obstructive jaundice in small cell lung cancer

**References**	**Gender**	**Age**	**Side of primary tumour**	**Location of secondary tumour**	**Pattern of metastasis**	**Other metastases**	**Treatment**	**Response to chemotherapy**	**Survival**
Smith HJ.^30^	NA	NA	NA	NA	NA	Present	Supportive	NA	4 weeks
Smith HJ.^30^	NA	NA	NA	NA	NA	Present	cholecystojejunostomy	NA	10 weeks
Smith HJ.^30^	NA	Na	NA	Na	NA	Present	Supportive	NA	<2 weeks
Jeong IB *et al.*^34^	Female	65	Left	Head	multiple	Absent	Percutaneous transhepatic biliary drainage following chemotherapy	Complete remission	>11 months
Howe HR *et al.*^35^	Female	40	Right	Head	solitary	Present	Choledochojejunostomy, irradiation, chemotherapy	Minimal response	1 month
Howe HR *et al.*^35^	Male	53	Right	Head	solitary	Present	Gastrojejunostomy + cholecystojejunostomy	NA	<2 weeks
Obara M *et al.*^40^	Male	69	Left	Head	multiple	Present	Percutaneous transhepatic biliary drainage followed chemotherapy	Partial remission	10 months
Kotan C *et al.*^41^	Male	46	Right	Head	solitary	Present	Pancreaticoduodenectomy followed chemotherapy	NA	11 months
Sakar A *et al.*^42^	Male	64	Right	Head	solitary	Absent	Gastrojejunostomy + cholecystojejunostomy	NA	<2 weeks
Singh D *et al.*^43^	Female	50	Right	NA	multiple	Absent	Endoscopic retrograde cholangiopancreatography	NA	NA
Ochi N *et al.*^44^	Male	64	Left	Head	solitary	Absent	Endoscopic retrograde biliary drainage, chemotherapy	Partial remission	25 months
Ochi N *et al.*^44^	Male	74	Left	Head	solitary	Present	Endoscopic retrograde biliary drainage, chemotherapy	Partial remission	4 months
Dunkerley RC and Dunn GD^45^	Male	72	Left	Head	solitary	Present	Endoscopic retrograde biliary drainage, radiotherapy	NA	6 months

NA = not available

**TABLE 2.** The characteristics of the patients with metastasis-induced acute pancreatitis in small cell lung cancer

**References**	**Gender**	**Age**	**Side of primary tumor**	**Location of secondary tumor**	**Pattern of metastasis**	**Tissue diagnosis**	**Other metastases**	**Treatment**	**Response to chemotherapy**	**Survival**
Woo JS *et al.*^33^	Female	45	Left	Head	multiple	No	Yes	Endoscopic stenting	NA	NA
Liu SF *et al.*^36^	Male	44	NA	NA	NA	Yes	Yes	Chemotherapy	NA	7.6 months
Liu SF *et al.*^36^	Female	44	NA	NA	NA	Yes	Yes	Chemotherapy	NA	3.2 months
Liu SF *et al.*^36^	Male	51	NA	NA	NA	Yes	Yes	Chemotherapy	NA	9 months
Liu SF *et al.*^36^	Male	75	NA	NA	NA	Yes	Yes	Chemotherapy	NA	1 month
Liu SF *et al.*^36^	Female	68	NA	NA	NA	Yes	Yes	Conservative	NA	2.7 months
Kim KH *et al.*^46^	Male	63	Right	NA	multiple	No	Yes	Conservative	NA	NA
Chowhan NM *et al.*^47^	Male	55	Left	Tail	NA	No	Yes	Conservative	NA	2 weeks
Chowhan NM *et al.*^47^	Male	48	Right	Head	Solitary	No	Yes	Conservative	NA	2 weeks
Levine M *et al.*^48^	Male	45	Right	All	NA	Autopsy	Yes	Conservative	NA	1 month
Yeung KY *et al.*^49^	Male	53	Left	Head and body	Diffuse enlargement	Autopsy	Yes	Chemotherapy	Partial remission	5.5 months
Yeung KY *et al.*^49^	Male	53	Right	NA	NA	Autopsy	Yes	Chemotherapy	Partial remission	5.5 months
Yeung KY *et al.*^49^	Female	37	Right	NA	Diffuse enlargement	No	No	Chemotherapy	Complete remission	3.5 months
Stewart KC *et al.*^50^	Female	44	Left	Head	Solitary	No	Yes	Chemotherapy	Partial remission	>6 months
Papagiannis A *et al.*^51^	Male	52	Left	NA	Diffuse enlargement	No	?	Conservative	NA	3 weeks
Allan SG *et al.*^52^	Female	73	Left	NA	Diffuse enlargement	No	Yes	Conservative	NA	5 days
Allan SG *et al.*^52^	Male	67	NA	NA	Diffuse enlargement	No malignity	Yes	Conservative	NA	10 days
Huang YW *et al.*^53^	Female	68	Left	NA	Diffuse enlargement	No	Yes	Conservative	NA	9 days
Hall M *et al.*^54^	Male	67	Left	Head	NA	Autopsy	Yes	Conservative	NA	2 weeks
Wurm Johansson G *et al.*^55^	Female	52	NA	Head and body	multiple	EUS-FNA	NA	Biliary stent + chemotherapy	NA	NA
Tanaka H *et al.*^56^	Female	51	Left	Body and tail	multiple	No	Yes	Chemotherapy	Complete remission	8 months
Noseda A *et al.*^57^	Male	58	Left	Head	Solitary	No	Yes	Conservative	NA	7 weeks
Schmitt JK^58^	Male	58	Left	NA	NA	Autopsy	Yes	Chemotherapy	NA	6.5 weeks
Evans AT^59^	Female	45	Right	NA	Diffuse enlargement	Autopsy	Yes	Conservative	NA	8 days
Chao WC *et al.*^60^	Female	65	Left	NA	multiple	No	Yes	Conservative	NA	20 days
Maclennan AC *et al.*^61^	Female	56	Right	All	multiple	Autopsy	?	Conservative	NA	5 weeks

NA = not available; EUS-FNA = ultrasound-guided fine-needle aspiration

**TABLE 3.** Pattern of metastasis in small cell lung cancer

**References**	**Gender**	**Age**	**Location**	**Type**	**Other metastases**
Sato M *et al*.^7^	male	78	body	solitary	Yes
Sato M *et al*.^7^	male	58	NA	NA	Yes
Sato M *et al*.^7^	male	75	NA	NA	Yes
Muranaka T *et al*.^10^	male	60	NA	Diffuse enlargement	NA
Volmar KE *et al*.^16^	male	58	NA	NA	No
Volmar KE *et al*.^16^	male	65	NA	NA	No
Mesa H *et al*.^18^	NA	NA	body	NA	NA
Gilbert CM *et al*.^19^	male	65	head	multiple	NA
Gilbert CM *et al*.^19^	male	58	head	solitary	NA
Gilbert CM *et al*.^19^	female	58	body	solitary	NA
Layfield LJ *et al*.^21^	female	57	head	NA	NA
Tsitouridis I *et al*.^24^	male	69	Head, body, tail	multiple	Yes
Tsitouridis I *et al*.^24^	male	59	Head and tail	multiple	yes
DeWitt J *et al*.^32^	female	49	head	solitary	Yes
DeWitt J *et al*.^32^	female	75	head	solitary	Yes
DeWitt J *et al*.^32^	male	49	head	solitary	NA
DeWitt J *et al*.^32^	male	65	head	solitary	NA
Scatarige JC *et al*.^37^	male	48	head	solitary	Yes
Scatarige JC *et al*.^37^	female	68	Head and body	multiple	NA
Xi-wen S *et al*.^38^	male	74	body	solitary	Yes
Xi-wen S *et al*.^38^	male	50	NA	solitary	Yes
Xi-wen S *et al*.^38^	male	68	NA	solitary	Yes
Xi-wen S *et al*.^38^	male	52	NA	solitary	Yes
Xi-wen S *et al*.^38^	female	39	NA	solitary	Yes
Xi-wen S *et al*.^38^	male	53	NA	solitary	Yes
Xi-wen S *et al*.^38^	male	53	NA	multiple	Yes
Xi-wen S *et al*.^38^	male	76	NA	solitary	yes
Katsuura Y *et al*.^39^	male	83	body	solitary	Yes
Merkle EM *et al*.^62^	male	55	tail	solitary	NA
Das DK *et al*.^63^	male	66	tail	solitary	NA
Walshe T *et al*.^64^	female	60	neck	solitary	No
Boo SJ *et al*.^65^	male	44	head	NA	No
Boo SJ *et al*.^65^	male	75	head	NA	No
Boo SJ *et al*.^65^	male	61	head	NA	Yes
Ottaiano A *et al*.^66^	male	65	Head, body, tail	NA	No

NA = not available
